# Are the Bacteria and Their Metabolites Contributing for Gut Inflammation on GSD-Ia Patients?

**DOI:** 10.3390/metabo12090873

**Published:** 2022-09-16

**Authors:** Karina Colonetti, Evelise Leis de Carvalho, Darlene Lopes Rangel, Paulo Marcos Pinto, Luiz Fernando Wurdig Roesch, Franciele Cabral Pinheiro, Ida Vanessa Doederlein Schwartz

**Affiliations:** 1 Laboratory of Basic Research and Advanced Investigations in Neurosciences (BRAIN), Porto Alegre 90035-903, RS, Brazil; 2 Laboratório de Proteômica Aplicada (LPA), Universidade Federal do Pampa, São Gabriel 97300-000, RS, Brazil; 3 Department of Microbiology and Cell Science, Institute of Food and Agricultural Sciences, University of Florida, Gainesville 32611, FL, USA; 4 Laboratório de Biologia, Universidade Federal do Pampa, Itaqui 97650-000, RS, Brazil; 5 Post-Graduate Program in Genetics and Molecular Biology, Universidade Federal do Rio Grande do Sul, Porto Alegre 91501-970, RS, Brazil; 6 Medical Genetics Service, Hospital de Clínicas de Porto Alegre, Porto Alegre 90035-903, RS, Brazil

**Keywords:** glycogen storage disease, inflammation, short-chain fatty acids, gut microbiota, dysbiosis, fecal pH

## Abstract

Recently, patients with glycogen storage disease (GSD) have been described as having gut dysbiosis, lower fecal pH, and an imbalance in SCFAs due to an increase in acetate and propionate levels. Here, we report the fecal measurement of bacterial-related metabolites formic, acetic, lactic, propionic, and succinic acid, a key metabolite of both host and microbiota, on a previously described cohort of 24 patients (GSD Ia = 15, GSD Ib = 5, 1 GSD III = 1 and GSD IX = 3) and 16 healthy controls, with similar sex and age, using the high-performance liquid chromatography technique. The succinic acid levels were higher in the GSD patients than in the controls (patients = 38.02; controls = 27.53; *p* = 0.045), without differences between the groups for other metabolites. Fecal pH present inverse correlation with lactic acid (R = −0.54; *p* = 0.0085), while OTUs were inversely correlated with both lactic (R = −0.46; *p* = 0.026) and formic (R = −0.54; *p* = 0.026) acids. Using two distinct metrics of diversity, borderline significance was obtained for propionic acid, affecting the microbial structure on Euclidean basis in 8% (r^2^ = 0.081; *p* = 0.079), and for lactic acid, affecting 6% of microbial structure using Bray–Curtis distance (r^2^ = 0.065; *p* = 0.060). No correlation was found between SCFAs and total carbohydrate consumption among the participants or uncooked cornstarch consumption among the patients.

## 1. Introduction

Hepatic glycogen storage diseases (GSD) are genetic diseases treated mainly by dietetic management, with simple sugar restriction and an overload of uncooked cornstarch (UCCS) [1,2]. There are several types of GSD, but GSD type I is the most frequent and severe in terms of UCCS dosage, and patients frequently present as overweight. GSD type I is classified as GSD Ia (OMIM#232200) or GSD Ib (OMIM#232220), and both are the result of the impairment of the glucose-6-phosphatase complex (G6PC). While GSD Ia affects one catalytic subunit of the complex (G6PC1 gene), GSD Ib affects the transporter protein on the endoplasmic reticle membrane (SLC37A4 gene). Once the transporter is affected, the whole complex will be affected due to the restriction of substrate availability [3]. Clinically, these patients also will be different since GSD Ib patients have a classical immunological impairment and inflammatory bowel disease (IBD) [4,5]. Despite not being a classic feature, IBD has also been described in GSD Ia patients, and the alteration of the gut microbiome because of the massive intake of UCCS may play a role in that [6,7]. Host intestinal health depends on the intestinal homeostasis between the innate/adaptive immune system and the microbiome. Numerous studies suggest that gut microbiota is constantly monitored by the host mucosal immune system, and any slight disturbance in the microbial communities may contribute to intestinal immune disruption and increased susceptibility to IBD, a chronic relapsing inflammatory condition of the gastrointestinal tract [8].

Fecal short-chain fatty acids (SCFA) and small molecules such as succinate are products of microbial fermentation in the large bowel from food components unabsorbed/undigested in the small intestine; they are characterized by containing fewer than six carbons (C), existing in straight, and branched-chain conformation. Acetic acid (C2), propionic acid (C3), and butyric acid (C4) are the most abundant, representing 90–95% of the SCFA present in the colon [9]. Those metabolites exert a biological role over the host and are also capable of interfering with bacterial community fitness. Their production is largely influenced by pH and the available substrates [10,11]. On the other hand, the types of SCFAs produced influence the luminal pH [12]. In addition to that, the luminal pH in the colon is modulated by mucosal bicarbonate, lactate production, the bacterial fermentation of carbohydrates, and the mucosal absorption of SCFA [13]. The association between these factors and lower pH, SCFA and gut inflammation has been reported by several studies [14,15].

The bacterial species, their metabolic products, and the host immune system may influence whether host cellular homeostasis is maintained or inflammatory mechanisms are triggered. Our previous study [16] demonstrated that GSD patients presented low fecal pH and high calprotectin levels, in addition to intestinal dysbiosis, with increased Proteobacteria, recently confirmed by Ceccarani and colleagues [17]. Therefore, the aim of this study was to analyze how these aspects influence dysbiosis in patients with GSD, which was previously described. Thus, we report the quantification of fecal formic, acetic, lactic, propionic, and succinic acid of a previous well-characterized cohort of patients described in our aforementioned study and their association with fecal pH, microbial diversity, diet, and calprotectin levels found in the patients and controls.

## 2. Materials and Methods

This was an observational, cross-sectional, controlled study with convenience sampling. Twenty-four GSD patients (GSD Ia = 15, GSD Ib = 5, 1 GSD III = 1, and GSD IX = 3) receiving UCCS treatment were recruited from the outpatient clinics of the Medical Genetics Service at Hospital de Clínicas de Porto Alegre (MGS-HCPA), Brazil from January 2016 to May 2017, as previously described [16]. Briefly, as inclusion criteria, the subjects (patients and controls) should be ≥3 years old, have no signs of infection, have not been vaccinated for at least 15 days prior to sample collection, not be taking antibiotics, nor should they have been designated to receive/received an organ transplant. The healthy controls (*n* = 16) were recruited by invitation as they came to routine appointments at Santa Cecília Basic Health Unit, Porto Alegre, Brazil. The minimum–maximum age of the included patients and controls were 10–19.75 and 10–23.25 years, respectively. All of the subjects received a kit and printed instructions for their own stool collection, as well as storage and transport. Upon returning to the clinic, the fecal samples were frozen at −80 °C until use.

### 2.1. Sample Preparation and Fecal Short Chain Fatty Acids Measurement

To determine the SCFAs in the feces, 150 mg of each of the frozen fecal samples from the patients and controls was aliquoted at room temperature (20 °C) and homogenized in 1.5 mL H_2_SO_4_ 0.15 mM for 2 min. Then, the samples were centrifuged for 5 min at 10,000× *g* rpm/4 °C. The supernatant was removed, filtered on a 0.22 µm pore nitrocellulose membrane, and frozen at −20 °C until analysis on a high-performance liquid chromatography (HPLC) system.

Commercial standards of acetic (AA), formic (FA), lactic (LA), succinic (SA), and propionic (PA) acid were used to obtain the calibration curve and thus quantify the SCFAs using the HPLC system (Shimadzu Prominence® UFLC, LC-6AD pump, SPD-20AV UV detector; Shimadzu, Kyoto, Japan).

Separation was achieved on a Nucleosil 100-5 C18 EC (d.i. 250 mm × 4.6 mm, 100 Å e 5.0 µm; Macherey-Nagel, Dueren, Germany) HPLC column attached to a pre-column Security Guard Cartridge System (Phenomenex, Torrance, USA) using monosodium phosphate solution (20 mM; pH 2.2) (A) and acetonitrile (B) and an isocratic mode 95:5 with a flow rate of 1.25 mL/min. The injection volume was 20 μL. The chromatograms were monitored at 210 nm. The LC Solution software (Shimadzu) was used to obtain the retention time and the chromatograms and to perform the quantification of the peak area. Protocol standardization was performed using triplicate samples.

The quantification of SCFAs in the fecal sample was performed according to De Baere et al. (2013) [18], with some modifications. The samples were compared with a mix of standard SCFAs and succinic acid, composed of FA, LA, AA, SA, and PA, with degrees of purity ranging from 85 to 99.9%. The working solution (5 M) was prepared, and serial dilutions (200–0.048 mM) were performed to obtain the retention time of the fatty acids (AA = 1.739 min; FA = 2.718 min; LA = 3.247 min; PA = 4.302 min; SA = 4,462 min) and the calibration curves [AA y = 26,960x − 31,217 (r = 0.9846); FA y = 23,042x + 25,601 (r = 0.9982); LA y = 27,648x − 60,305 (r = 0.9714); PA y = 35,066x + 86,765 (r = 0.9911); SA y = 12,766x − 289,128 (r = 0.9631)].

The limit of detection (LoD) and limit of quantification (LoQ) for each metabolite were performed: FA LoD = 0.024 mM, LoQ = 0.048 mM; LA LoD = 0.048 mM, LoQ = 0.195 mM; AA LoD = 0.195 mM, LoQ = 0.390 mM; SA LoD = 0.098 mM, LoQ = 0.390 mM; PA LoD = 0.098 mM; LoQ = 0.195 mM.

### 2.2. Statistical Analysis

Statistical analysis among the groups was performed using PASW Statistics for Windows software (Vs18.0, 2009, SPSS Inc., Chicago, IL, USA) and R Studio software Version 1.3.959, using the phyloseq [19] and vegan [20] packages after dataset rarefaction to the minimum library size [20,21]. The numerical variables were compared using the Mann–Whitney U test. The categorical variables were compared using X^2^, Fisher’s exact test, or Continuity Correction, when necessary (statistically significant results were determined by the threshold *p* ≤ 0.05). The graphs were constructed in R Studio software. To analyze the relationship between calprotectin and SCFAs, the valid reads of these last ones were used as the limit of the points on the graph. The valid reads of SCFAs were plotted on the Y-axis.

## 3. Results

### 3.1. Cohort Description, Metabolite Quantification and Differences on Microbial Community between Patients and Controls

The clinical characteristics, food intake, and gut microbial profile of the 24 patients and 16 controls that comprised this study were previously described in detail, as well as the features of the gut microbiota [16].

**Figure 1:**
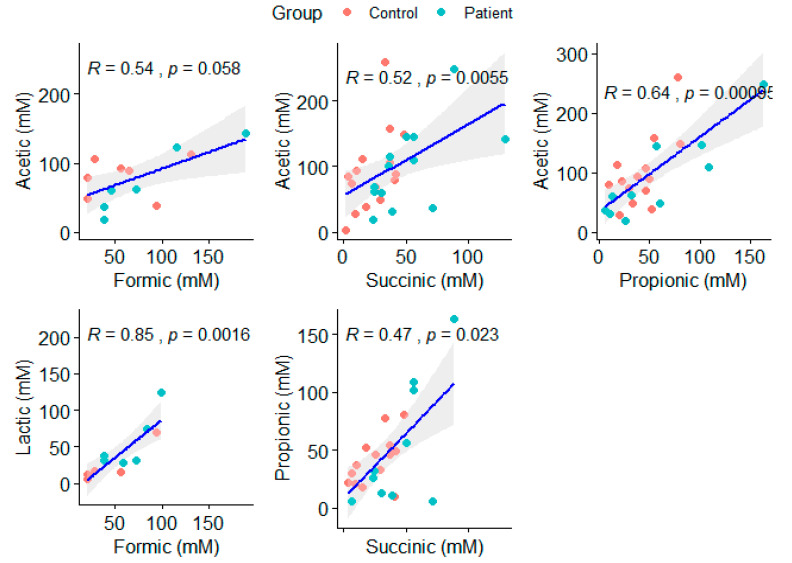
Significative correlations (*p* > 0.05) between acetic, propionic, succinic, lactic and formic acids. As the acetic and formic acids has a related metabolic pathway, this correlation is also shown above although it is not significant.

Using the set of data with patients and controls, formic (FA), acetic (AA), lactic (LA), propionic (PA), and succinic (SA) acids were compared between the groups (Table 1). Among all of the metabolites, only SA was present on different levels, and was higher in patients (patients = 38.02, controls = 27.53; *p* = 0.045). However, taking the characteristics of the microbial community into account, none of the metabolites isolated were directly related to alterations to the microbial structure at the significance level threshold (*p* = 0.05), with LA presenting borderline significance (*p* = 0.06) and the effect size over the microbial structure of 6.5%. Considering the fact that SA was different between the groups, the borderline significance of some metabolites, and the correlation among AA and SA (r = 0.52; *p* = 0.0055); AA and PA (r = 0.64; *p* = 0.00095), FA and LA (r = 0.85; *p* = 0.0016); and PA and SA (r = 0.47; *p* = 0.023) (Figure 1), we also inquire about the combined effect of these metabolites on the microbial structure found in the participants. SA seems to be an important adjuvant for both LA and PA effects on the microbial structure, increasing the effect size to 33% and 11%, respectively, both with statistical significance (*p* < 0.05) (Table 1).

### 3.2. Metabolites, Fecal pH and Observed Operational Taxonomic Units

**Figure 2:**
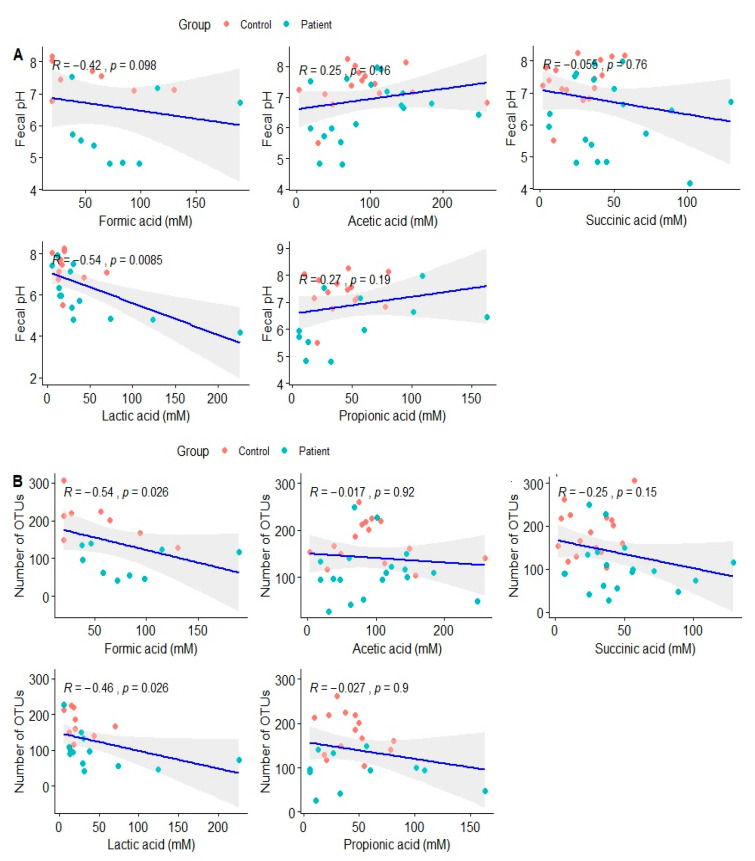
Panel show correlations of acetic, propionic, succinic, lactic and formic acids with (**A**) fecal pH; and (**B**) Observed OTUs between OTUs in fecal samples of patients and controls.

As second step, tests were performed to determine if there were correlations between each metabolite and the fecal pH (Figure 2A) and also between the metabolites and the number of operational taxonomic units observed on subjects (Figure 2B). Microbial diversity was found decreased when FA (r = −0.54; *p* = 0.026) and LA (r = −0.46; *p* = 0.026) are high, while low fecal pH is associated with higher levels of LA (r = −0.54; *p* = 0.0085). Lactic and formic acid were found to be strongly correlated between them (r = 0.85; *p* = 0.0016).

**Figure 3:**
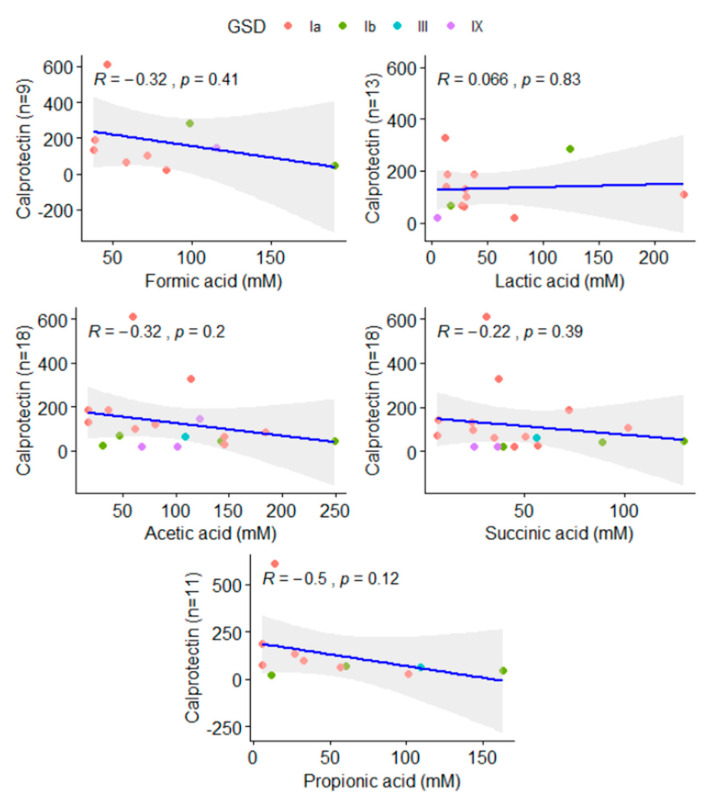
Correlation of acetic, propionic, succinic, lactic, and formic acids and calprotectin levels found in patients. The number of valid reads available in each comparison was added to Y-axis.

No variation was found among the metabolites when GSD Ia and GSD Ib patients were compared. The number of valid observations did not allow a comparison with GSD type III and IX (Table 2). Metabolites were not directly related to the calprotectin levels found on patients (Figure 3). Additionally, the relationship between BMI and SCFAs was analyzed (data not shown), but no statistically significant difference was observed.

## 4. Discussion

The pathophysiology and gut microbial alteration during inflammatory bowel disease in GSD patients are still not completely understood. In our previous study, we described the biochemical alterations in the gut environment of GSD patients, such as acidification and inflammation. Recently, propionate and acetate have been found to be increased in a group of nine GSD patients (Ia = 4; Ib = 5) and twelve healthy controls [17]. Instead, our study did not find propionate or acetate accumulation but an increase in succinic acid and a tendency towards formate accumulation in patients. Both studies are concordant regarding dysbiosis in GSD patients and the overrepresentation of Phylum Proteobacteria in patients, but they are not comparable due to factors previously mentioned by Ceccarani and colleagues, such as sequencing technologies and the reference database used for taxonomic classification. Additionally, fecal pH was not characterized by Ceccarani and colleagues (2020), as well as the relationships between metabolites and the structure of the microbial community, which are detailed in this study. This, among other factors already considered by Ceccarani and colleagues, might help to explain the different results in the microbial divergences at lower taxa levels found in the studies.

The profile of SCFAs is influenced by lifestyle, including physical activity and body composition [22]. However, it is known that diet is a major driver of the gut microbiome [23], and as previously published, this was found to explain the majority of differences between the patients and the controls [16]. SCFA formation represents the major flow of carbon from the diet through the microbiome to the host [15]. The principal SCFAs that results from both carbohydrate and amino acid fermentation are acetate, propionate, and butyrate, although formate and other metabolites are also produced in lesser amounts [24]. Other fermentation products such as lactate, ethanol, and succinate, which are intermediates in the global fermentation process in the microbiota, are to varying extents metabolized to SCFA by cross-feeding species in the ecosystem, and they do not usually accumulate in the bowel [25]. Some studies suggest that butyrogenic capacity can be related to lactate availability and that different gut microbial communities can metabolize lactate in different ways [26]. Furthermore, pH is a critical factor in fermentation reactions interfering with metabolite production [27], and thus, its role in metabolite production cannot be underestimated.

In this study, the metabolites did not interfere with the microbial structure in a statistically significant way when they were analyzed alone, but the number of samples was small, the gut is a complex environment, and a synergistic effect is more likely than an isolated effect. Table 1 shows that although some SCFAs can be statistically different between patients/controls, they are not able to affect the gut microbiome alone. The combined effect can be much more interesting when studied together. However, our technique seems not to be the most efficient one to study the SCFAs, given the low number of reads for some SCFAs, e.g., Formic and Propionic. Our number of patients/limited valid reads did not allow us to explore with confidence some trends in statistical patterns that arose from the data. Worthy of note, there was a difference between the patients and the controls for just one metabolite, SA. Although there was no difference in bacterial metabolite levels between the patients and controls for LA and PA, these metabolites alone reached borderline significance and had an interesting effect size on the microbial structure (6.5% and 8%, respectively). This effect was increased and became statistically significant when considering the sum of these metabolites and the amounts of SA, which were higher in patients than in controls (Table 1), indicating that SA has an important role in the gut environment.

SA was found to be increased in patients but had no impact on the microbial structure when considered apart. SA is an important metabolite in both host and microbial processes, playing a role in the activation of immune cells via SUCNR1, a G-protein coupled receptor, enhancing inflammation. Succinate accumulation is infrequently reported for human fecal samples, even in the presence of overweight [27]. However, SA is increased in dysbiosis, patients with IBD, and animal models of intestinal inflammation [28,29]. Previously, we have reported that calprotectin levels, a gut inflammation marker, were abnormal among 70% of GSD Ia patients. There was no correlation between calprotectin and SCFAs in this study (Figure 3).

Taking the biochemical aspects into account, SA is the precursor for propionate formation, but when sufficient carbohydrate is present, there is a reduced need to decarboxylate succinate, and this metabolite accumulates instead of propionate [30]. The UCCS used in the treatment of GSD patients is a mix of amylose and amylopectin, with15% of ileal effluents that leads to microbial fermentation in the colon [31] and might be related to the succinate levels found in the patients. Importantly, none of the metabolites alone or the tested sums correlated with the total carbohydrate intake among the participants or uncooked cornstarch consumption among the patients (data not shown).

Relatively little is known about the role of formate in the gut. Formic acid in the intestinal lumen has been reported as a product of microbial activity. It has been linked to methanogenesis and appears to be elevated in inflammatory conditions [32,33]. In vivo, formate served as an electron donor in conjunction with oxygen as the terminal electron acceptor. Formate oxidation and oxygen respiration have been described as metabolic signatures for inflammation-associated dysbiosis [34], thus consistent with reduced observed numbers of OUTs and dysbiosis and also with the depletion of methanogen previously reported in patients.

In this study, the levels of butyrate were not accessed. In addition, our technique was different from that used by Ceccarani and collaborators (HPLC vs. CG), and we are unable to verify if the pH conditions in the gut environment were similar among the subjects of this and the aforementioned study.

Here, we described an altered microbial metabolite profile on an SA basis, which are consistent with dysbiosis. Considering the limitations of this study, such as the limited data on gut inflammation and the absence of physical activity data in patients and controls, our results indicate that the association between IBD and the increased penetration of SCFAs [14] can be extended to patients with GSD. However, the mechanism underlying the immune activation of the gut environment in those patients remains unclear.

## Figures and Tables

**Table 1:** Metabolite quantification between patient and control group.

Metabolite ^1^	N ^2^ (Patient/Control)	Patient (mM) ^3^	Control (mM) ^3^	*p*-Value ^4^	Microbial Community Difference between Groups (r^2^; *p*-Value)	
					Euclidean	Bray-Curtis
Formic acid	09/08	71.74 (42.28–106.88)	41.82 (20.08–86.38)	0.149	0.057; 0.443	0.058; 0.523
Acetic acid	18/15	91.28 (44.96–143.20)	85.27 (48.75–112.19)	0.942	0.0276; 0.521	0.017; 0.989
Lactic acid	13/10	28.86 (13.91–56.14)	17.87 (13.08–25.78)	0.321	0.0562; 0.256	0.065; 0.060
Propionic acid	11/14	32.68 (11.37–10.51)	42.06 (22.06–52.98)	>0.999	0.081; 0.079	0.027; 0.905
Succinic acid	18/16	38.02 (24.60–60.33)	27.53 (9.50–39.99)	**0.045**	0.045; 0.131	0.034; 0.234
Formic + Succinic	7/8	61.28 (30.52–100.04)	37.89 (15.28–68.06)	0.224	0.054; 0.557	0.062; 0.627
Acetic + Succinic	13/15	101.59 (42.78–189.65)	89.31 (46.93–129.04)	0.440	0.067; 0.069	0.030; 0.701
Lactic + Succinic	10/10	49.18 (27.66–78.17)	35.28 (11.93–52.49)	0.061	0.073; 0.201	**0.332; 0.019**
Propionic + Succinic	10/14	45.17 (12.02–78.36)	50.91 (12.02–87.41)	0.564	**0.111; 0.030**	0.035; 0.688
Formic + Acetic	5/7	105.97 (51.83–188.98)	85.27 (41.10–151,36)	0.696	0.070; 0.465	0.079; 0.440
Propionic + Acetic	10/14	94.92 (14.56–189.54)	107.77 (64.65–134.86)	0.540	0.078; 0.087	0.223; 0.237
Formic + Lactic	6/5	46.03 (0–90.23)	27.49 (18.85–50.43)	0.714	0.099; 0.468	0.310; 0.054

^1^ Common name = IUPAC name: Formic acid = methanoic acid; Acetic acid = ethanoic acid; Lactic acid = 2-hydroxypropanoic acid; Propionic acid = Propanoic acid; Succinic acid = Butanedioic acid. ^2^ The number of patients analyzed changed between analyses; ^3^ Numeric variables were reported as medians (Q1–Q3); ^4^ Due to the not-normal distribution, numeric variables were subjected to the Mann–Whitney test. *p*-values < 0.05 were considered significant and are shown in bold.

**Table 2:** Description of clinical parameters and measured metabolites among GSD types.

Variable	Valid Observations GSD Ia/Ib ^1^	GSD Ia (*n* = 15) ^2^	GSD Ib (*n* = 5) ^3^	*p*-Value ^4^	GSD III (*n* = 1)	GSD IX ^3^ (*n* = 3)
Formic acid (methanoic acid)	06/02	52.00 (38.34–74.76)	98.50–189.58	NA	NA	115.26
Acetic acid (ethanoic acid)	10/04	71.68 (32.09–145.19)	95.15 (35.27–221.79)	0.777	109.25	68.28–122.67
Lactic acid (2-hydroxypropanoic acid)	10/02	29.78 (14.22–47.08)	16.71–124.19	NA	NA	5.34
Propionic acid (Propanoic acid)	07/03	26.45 (5.86–56.85)	11.37–162.97	0.305	109.10	-
Succinic acid (Butanedioic acid)	12/03	35.95 (23.75–54.95)	38.97–129.04	0.083	55.67	24.65–36.39

^1^ The number of patients analyzed changed between analyses; ^2^ Numeric variables were reported as medians (Q1–Q3); or ^3^ Min–Max for less than four observations; ^4^ Due to the not-normal distribution, numeric variables were subjected to the Mann–Whitney test. *p*-values < 0.05 were considered significant. NA = not accessed due to small number of valid observations. All GSD IX patients had valid measurements to ALT, AST, and acetic acid; lactic and formic acid obtained for one patient, and propionic acid had no reads.

## Data Availability

The data presented in this study are available in the main article.
